# Controlled Release of Tea Tree Oil from a Chitosan Matrix Containing Gold Nanoparticles

**DOI:** 10.3390/polym14183808

**Published:** 2022-09-12

**Authors:** Frederic Matussek, Adriana Pavinatto, Peggy Knospe, Sabine Beuermann, Rafaela Cristina Sanfelice

**Affiliations:** 1 Institute of Technical Chemistry, Clausthal University of Technology, Arnold-Sommerfeld Street 4, 38678 Clausthal-Zellerfeld, Germany; 2 Scientific and Technological Institute of Brazil University, Brazil University, 235 Carolina Fonseca Street, São Paulo 08230-030, SP, Brazil; 3 Institute of Particle Technology, Leibnizstraße 19, 38678 Clausthal-Zellerfeld, Germany; 4 Science and Technology Institute, Federal University of Alfenas, 11999 José Aurélio Vilela Road, BR 267, Km 533, Poços de Caldas 37715-400, MG, Brazil

**Keywords:** melaleuca oil, essential oil delivery, chitosan droplets, chitosan film, AuNPs

## Abstract

Chitosan is a biopolymer that, due to its versatile bioactive properties, has applications in several areas, including food, medicine and pharmaceuticals. In the field of tissue engineering, chitosan can be used, for example, as a dressing to treat wounds or dermal damage, such as burns or abrasions. This work deals with the controlled release of tea tree oil from chitosan-based polymeric films and droplets containing gold nanoparticles (AuNP). AuNPs were successfully incorporated into the chitosan matrix using two different approaches. Both solutions were loaded with tea tree oil, and from these solutions, it was possible to obtain drop-cast films and droplets. The controlled release of oil in water was performed both in the films and in the droplets. The addition of AuNP in the controlled release system of melaleuca oil favored a release time of around 25 h. A series of experiments was carried out to investigate the effects of different reaction temperatures and acetic acid concentrations on the formation of AuNPs in the presence of chitosan. For this purpose, images of the AuNP films and droplets were obtained using transmission electron microscopy. In addition, UV-vis spectra were recorded to investigate the release of tea tree oil from the different samples.

## 1. Introduction

Over the last century, the importance of polymer chemistry has had unprecedented growth. Due to their wide range of customizable properties, synthetic polymers have been commonly used in a large variety of different fields to fulfill a specific purpose. However, a common problem with these materials is their non-biodegradability. In this sense, recycling seems to be a good alternative to reduce waste; however, not all recycling processes are profitable and/or viable for all applications; for example, some recycled materials cannot be used for medical/health applications. Biopolymers, on the other hand, are naturally occurring polymers designed and optimized by nature itself, being mostly environmentally friendly. The rising popularity of green chemistry and environmentally friendly procedures and products have increased the demand for appropriate biopolymers or synthetic biodegradable polymers [1].

Among these products is chitin, a natural biopolymer that is mostly present as the major structural component in the exoskeletons of insects and crustaceans. It is a polysaccharide of β-(1-4) linked N-acetyl-D-glucosamine units, which can be N-deacetylated (usually exceeding 60 %) to obtain chitosan, which is soluble in diluted acidic solutions [2]. The excellent biocompatibility, non-toxic and non-immunogenic nature, biodegradability, antimicrobial activity, and accelerated wound healing properties have enabled chitosan to be used in various applications. Furthermore, chitosan can be used in agriculture as an elicitor to strengthen plant defenses and to react against certain pathogens to reduce lesions. In addition, seed treatment with chitosan can increase yield and promote plant growth. It is also a viable replacement for high-toxicity pesticides [3]. Another field of application is the food industry. Chitosan-based thin films can be effectively used as a protective layer for natural foods. For instance, Pavinatto et al. [4] reported that such films inhibited the formation of fungi and growth of bacteria while not affecting the flavor, aroma, and appearance of strawberries. In conclusion, due to the antimicrobial properties of chitosan, these films proved to be an efficient alternative to freezing, and are widely applied for the preservation of fresh foods [4]. Moreover, chitosan can be used in controlled-release studies. The release of molecules, such as drugs or essential oils in chitosan-based systems, occurs through polymer swelling, which allows the adsorbed molecules to be released by a diffusion process without polymer degradation [5,6].

The known antimicrobial activity of chitosan against Gram-positive, Gram-negative bacteria, and fungi is dependent on factors that include the type of microorganism, the pH of the medium, the structural characteristics of the polymer molecule (molecular mass, degree of acetylation), and the chitosan concentration in the medium [7]. Different mechanisms have been proposed to explain the action of chitosan and its derivatives against microorganisms. Currently, the most accepted hypothesis is that chitosan molecules alter the permeability of the microorganism cell membrane, preventing gas exchange between the inside and outside of the cell, which causes cellular dysfunction due to membrane rupture or penetration of molecules inside the cell, interfering with the synthesis of essential molecules such as DNA and RNA [7].

Chitosan has numerous medical applications as well, including tissue engineering, because of its versatile bioactive properties. It can be used to fabricate tissue substitutes in combination with living cells that can be implanted into the human body and skin [8]. It can also be used in dentistry as an adsorbent for certain oral bacteria to reduce the initial adsorption on the tooth surface or as an additive in dentifrices due to this behavior and its good antimicrobial properties [9]. Chitosan has positive effects on the acceleration of wound healing and the regeneration of damaged human skin because it facilitates the proliferation of fibroblasts and macrophages and, consequently, epithelium regeneration [10]. Due to these properties, it can be used as a dressing to treat wounds or dermal damage, such as burns or abrasions [11].

Among several studies involving the application of chitosan in the synthesis of gold nanoparticles, Huang and Yang were the first to report that chitosan has enhanced chelating characteristics compared to chitin due to the presence of primary amino groups, and it is commonly used as a reducing and stabilizing agent in the preparation of AuNPs for medical applications. Due to their biocompatibility and optical and electronic properties, AuNPs have applications in medicine [12,13]. For instance, surface-modified AuNPs have been used to improve the performance of diagnostics and the efficiency of drug delivery, which is enhanced due to cell penetration associated with the nanoscale size of nanoparticles, thus being highly recommended for cancer treatment [14,15]. In contrast to bulk gold material, gold nanomaterials have special applications in medicine and optical sensing because of their discontinuous surface electron energy levels [16]. The color of AuNPs, for example, is dependent on the electric surface charge density, which changes as a function of the size and shape of nanoparticles. In other words, larger particles lead to an absorption shift to longer wavelengths, resulting in a color change from red to blue. In addition to the change in color, the chemical reactivity also varies as a result of changes in the size and shape of the nanoparticles. Therefore, stabilizing agents can be specifically chosen to alter the solvation shell and fulfil the demands for a specific application [17].

The experiments on AuNP formation in this work are based on the sodium citrate method reported by Turkevich, Stevenson, and Hillier [18]. Since no organic solvents are necessary, the sodium citrate method is a green chemistry approach for AuNP formation. In the experiment, 95 mL of a solution containing 5 mL of hydrogen tetrachloroaurate (III) trihydrate is heated to the boiling point. Then, 5 mL of a 1% sodium citrate solution is added to the mixture. Good mechanical stirring is very important to support gold diffusion. Scientific advances involving the synthesis of gold nanoparticles have shown the effectiveness of their use in medical and food applications, thanks to their compatible biological effects [19]. In particular, the gold nanoparticles obtained by the Turckvich method are well accepted since their synthesis occurs in an aqueous medium using sodium citrate, which acts as a reducing agent of gold and a stabilizer of the nanoparticles. The gold salt solution is a yellowish color, but soon after the addition of the citrate solution, a color change is observed, indicating that the reaction has started. Sodium citrate acts both as a reducing agent and as stabilizing molecules for nanoparticles. When the gold salt is reduced to metallic gold, the gold atoms begin to agglomerate, causing the growth of the particles (bottom-up method). Citrate molecules, as stabilizers, are allocated around the growing nanoparticles, preventing uncontrolled growth. When the nanoparticles reach their size and shape, the solution acquires a reddish solution, which can have different shades depending on the size and shape of the particles. This method will be slightly modified for the purpose of this work so that chitosan acts as a stabilizing agent [20,21,22,23,24].

In this work, controlled release tests were carried out using tea tree oil [25,26,27,28,29,30,31,32] trapped inside the chitosan matrix containing gold nanoparticles. The tree oil can be extracted from a plant native to the Australian region called Melaleuca alternifolia by steam distillation processes of its leaves and branches [25,29]. Among its applications, tea tree oil is widely used to treat skin infections, as it has anti-inflammatory action and broad-spectrum antimicrobial activity against Gram-positive and Gram-negative bacteria and some types of viruses. Terpinen-4-ol is one of the main active components of tea tree extract and is primarily responsible for its antimicrobial action [32]. Due to its high volatility and ease of oxidation under ambient conditions with respect to light, temperature, and oxygen, it is of great interest to incorporate terpinen-4-ol into matrices consisting mainly of natural polymers, thus protecting it from natural degradation whilst controlling its release to the desired environment [29].

## 2. Materials and Methods

### 2.1. Materials

Tea tree oil was purchased from LDREAMAM^R^ (Anqing, China) and used as received (International Standard ISO 4730). Chitosan medium molecular weight (CAS Number 9012-76-4), glycerol (CAS number 56-81-5), acetic acid (CAS number 64-19-7), triphenyl phosphate (TPP) (CAS number 115-86-6), sodium citrate (CAS number 18996-35-5), hydrogen tetrachloroaurate (III) trihydrate (30 wt. % in dilute HCl) (CAS number 16961-25-4), and Tween 80 (CAS number 9005-65-5) were purchased from Sigma-Aldrich (Darmstadt, Germany) and also used as received. 

### 2.2. AuNP Formation at Different Temperatures and Acetic Acid Concentrations

Acetic acid was used as a reducing agent for gold nanoparticles because it is a favorable medium for chitosan solubilization [33,34]. To study the influence of acetic acid concentration and reaction temperature on AuNPs formed in a chitosan solution, a series of nine experiments were carried out. Table 1 shows the different samples and their respective parameters. Three different acetic acid concentrations (0.5, 1, and 2%) and three different temperatures (room temperature around 25 °C (RT), 40 °C, and 60 °C) were used in the experiments. 

Three stock solutions of 2% (*w*/*w*) chitosan with different proportions of acetic acid were prepared by stirring constantly at room temperature for 12 h. The concentration of acetic acid of each solution was as follows: solution (1)—4%; solution (2)—2%; and solution (3)—1%. Solution (4) was a gold salt solution containing 34 μL of hydrogen tetrachloroaurate (III) trihydrate in 100 mL of pure water. 

For the first experiment, 10 mL of solution (1) was mixed with 10 mL of solution (4) at room temperature under continuous stirring for 32 h to form the AuNPs. To determine the effect of acid concentration on the AuNPs, the same experiment was performed with solutions (2) and (3) as replacements for solution (1). To understand the influence of the reaction temperature on the AuNPs, the experiments were repeated at 40 °C and 60 °C, and all of them were performed in triplicate. A water bath was used to heat 10 mL of the chitosan solution and solution (4) separately to the desired temperature. As soon as the temperature was reached, 10 mL of solution (4) was added to the chitosan solution under continuous stirring up to 10 min after the color change. 

### 2.3. Synthesis of Gold Nanoparticles with Sodium Citrate

To minimize chemical waste as well as biological and environmental threats and to support further advancement in the green chemistry procedures, the AuNP were synthesized according to a modified version of the Turkevich method [35,36,37], in which water was used as the solvent and sodium citrate was used as both the reducing and the stabilizing agent. Subsequently, sodium citrate was mixed with chitosan to combine the biopolymer matrix with gold nanoparticles.

**AuNP/Citrate (AuNP#10):** 2 g of sodium citrate was dissolved in 98 mL of pure water to create a 2% sodium citrate solution. Then, 25 mL of this solution was added to 100 mL of pure water. The solution was then heated to the boiling point of water under continuous stirring using an oil bath. At this point, 34 μL of hydrogen tetrachloroaurate (III) trihydrate (HAuCl) was added, and a change in color was observed after a few minutes: the solution turned deep red and showed no precipitate. The stirring and heating were maintained for another 10 min to stabilize the nanoparticles. The remaining solution was used to measure the UV-vis (300–700 nm) spectrum using a UVIKON XS/XL spectrometer (SCHOTT Instruments—Mainz, Germany).

**AuNP/Citrate + Chitosan (AuNP#11):** 50 mL of a 2% (*w*/*w*) chitosan stock solution was mixed with 50 mL of a synthesized gold nanoparticle solution. The UV-vis spectrum was measured for comparison with the other solutions.

### 2.4. TEM Images

The gold nanoparticle morphology was observed using a transmission electron microscope (TEM, JEM 2100, Jeol, Sao Paulo, Brazil) operated at 120 kV located at the Institute of Particle Technology (TU-Clausthal). The gold nanoparticles were deposited on a carbon-coated copper grid.

### 2.5. Formation of Chitosan-Based Films

The solutions of AuNP#9 and AuNP#11 were used to create the polymeric films due to the similarity in the shape and size of NPs to those reported by Turkvic et al. [37]. To this end, 0.5 g of glycerol was added to both solutions with constant stirring for 10 min to increase the flexibility and mechanical stability of the films since glycerol acts as a plasticizer agent of chitosan molecules. Subsequently, 0.1 g of polysorbate 80 and 0.1 g of tea tree oil (approximately nine drops) were added to the solution. The polysorbate acts as a surfactant to mix the oil with the aqueous solution, helping to embed it into the chitosan matrix. Afterward, 6 mL of both solutions were put into different silicone molds (3 cm in diameter), which were left in an oven at 40 °C overnight. The UV-Vis absorption bands of the oil before being added to the film and those observed during release showed the same maximum absorption value. Therefore, it is suggested that there was no change in the integrity of the oil during this period.

### 2.6. Formation of Chitosan-Based Droplets

In the AuNP#9 and AuNP#11 solutions, tea tree oil was added with the help of the Tween 80 surfactant to promote complete solubilization. These solutions were also used to create polymer droplets. In a separate beaker, a solution containing TPP and sodium citrate was prepared. For that, 0.7 g of TPP and 0.3 g of sodium citrate were dissolved in 100 mL of pure water, and the solution was kept under stirring for 1 h. Afterward, a petri dish was filled with 10 mL of this TPP solution. A syringe was used to take 6 mL of the chitosan solution, which was then slowly added dropwise to the TPP solution. After 30 min, the TPP solution was replaced with pure water, which was removed after another 30 min. The droplets were then left in an oven at 40 °C overnight.

### 2.7. Controlled Release Experiment

The setup for the controlled release experiment using either droplets or films was similar. An equivalent droplet and film mass (about 0.10 g) were placed into a beaker containing 30 mL of pure water under continuous weak stirring. In the beginning, the UV-vis spectra were measured every 10 min using approximately 2 mL of the beaker solution, and this solution was placed back in the beaker post-measurement. After 120 min, the time between measurements was increased to 30 min. The experiment ended after 25 h and 30 min. The equilibrium was reached, and there was no further increase in absorption in the spectra (from oil concentration).

The process of tea tree oil release from a chitosan-based polymer matrix follows a diffusion-controlled model based on Fick’s second law of diffusion, as was reported in the paper of Fu et al. [5]. The authors suggested that this model can be applied to drug releases in the case of slab-like devices. In the present work, this model is not well represented in the case of the film prepared from the AuNP#9 solution, as will be described in the Section 3. In the other cases, Equation (1) describes the long-time approximation of the drug release:

(1) $$\frac{M_{t}}{M_{0}}=1-\left(\frac{8}{\pi^{2}}\right)\exp\left(\frac{-\pi^{2}Dt}{h^{2}}\right),$$

where $M_{t}$ is the amount of drug released at time t, $M_{0}$ is the initial amount of drug loaded into the carrier, D is the diffusion coefficient of the drug within the polymer matrix, and h is the thickness of the carrier device (average value among 3 measurements). This equation can be compared to that obtained for an exponential curve, characteristic of controlled release processes. The term ($\frac{\pi^{2}D}{h^{2}}$) from Equation (1) resembles *K*, used for an exponential curve given in the general form of *A= c* − exp *(−Kt)*. The actual fit to the data is provided in the figures. Knowing h from measurement of the initial dry film, the diffusion coefficient *D* can be derived from the experimental data. 

## 3. Results and Discussion

### 3.1. AuNP Formation at Different Temperatures and Acetic Acid Concentrations

**Figure 1:**
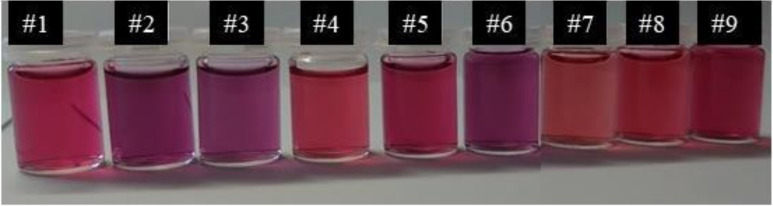
AuNP solutions synthesized by varying the acetic acid concentration and temperature according to the conditions reported in Table 1.

In order to evaluate the influence of the temperature and acetic acid concentration on the formation of AuNPs containing chitosan, nine different syntheses were carried out. Figure 1 illustrates the different solutions created to compare the AuNPs prepared with different acetic acid concentrations and at different reaction temperatures. The numbers refer to the sample numbers used in Table 1. Figure 1 clearly shows that both the temperature and acetic acid concentration influence the color of the AuNP solutions and, consequently, their size and shape. The more intense the reddish color, the smaller the value of the wavelength of maximum absorption and, consequently, the smaller the particles formed.

**Figure 2:**
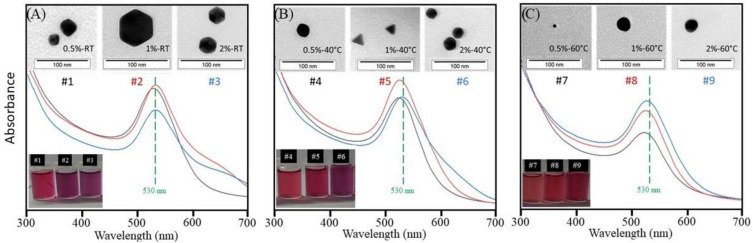
UV-vis spectra of AuNP solutions. In the inset: images of solutions and TEM-images of nanoparticles formed: (**A**) at room temperature and acetic acid concentration of 0.5, 1, and 2%; (**B**) at 40 °C and acetic acid concentration of 0.5, 1, and 2%; and (**C**) at 60 °C and acetic acid concentration of 0.5, 1, and 2%.

Figure 2A displays the UV-vis spectra of samples AuNP#1, AuNP#2, and AuNP#3, which are the three solutions prepared at room temperature with acetic acid concentrations of 0.5, 1, and 2%, respectively. It can be seen that the absorbance maxima change slightly to higher wavelengths with increasing acid concentration, staying around 530 nm. The bandwidths also become slightly larger with increasing acid concentration. By comparing the color of the three solutions, a change from red to purple is clearly visible with increasing acid concentration, as seen in the lower inset of Figure 2A. Therefore, both the UV-vis spectra and the color change of the solutions indicate that the polydispersity and/or shape of AuNPs are influenced by the concentration of acetic acid present in the solution. The shape and size of the nanoparticles were analyzed by transmission electron microscopy (TEM), and the images are also illustrated in the inset of Figure 2A. The TEM images suggest that in all cases, nanoparticles with a hexagonal shape, with a diagonal varying between 20 and 50 nm, were obtained.

Figure 2B,C display the UV-vis spectra of the solutions prepared at 40 °C and 60 °C, respectively. It can be noted that both exhibit similar behavior to the solutions in Figure 2A in relation to bandwidths and absorbance maxima. However, the differences become weaker with increasing temperature, as clearly seen in Figure 2C. In addition, although a color change can also be observed for the temperature sets created at 40 °C and 60 °C, the variation in color decreases as the temperature increases. This is also visible in the UV-vis spectra when comparing the differences in bandwidths and wavelength of absorbance maxima of AuNP solutions (Figure 2A–C).

The TEM images of the nanoparticles synthesized at a temperature of 40 °C (inset of Figure 2B) show a significant variation in the shape of the particles formed when the acid concentration was varied. At a concentration of 0.5%, irregularly-shaped particles (as if the particle was between a spherical shape and a hexagonal shape) were observed throughout the entire sample. The samples obtained with an acid concentration of 1% and 2% presented triangular and hexagonal nanoparticles, respectively, sized between 10 and 20 nm. For the nanoparticles obtained at a temperature of 60 °C (inset of Figure 2C), it was observed that the shape of the particles was predominantly spherical, with a diameter between 5 and 20 nm. The impact of the process conditions on the shape of the gold nanoparticles is in agreement with previous reports [38,39].

It can be concluded that a change in the system temperature considerably modified the shape of the gold nanoparticles formed in the presence of chitosan. Specifically, in the case of nanoparticles obtained at a temperature of 40 °C, it was verified that the acetic acid concentration also influenced their shape. Regarding the particle size, it varied as a function of acid concentration and temperature.

The AuNP#9 solution was chosen to be used in studies involving the controlled release of tea tree oil, and for comparison purposes, the matrices were also used from the mixture of a 2% chitosan solution and gold nanoparticles (AuNP#11) obtained by the method from Enustun et al. This was the solution chosen due to the similarity in shape and size of nanoparticles with those obtained by the Turkvic method [36]. It is possible to find in the literature the synthesis of gold nanoparticles using different reducing agents and different stabilizers [12,36,40,41,42]. One of the most used methods for the synthesis of gold nanoparticles is the Turkvich method, in which sodium citrate is used both as a reducing and stabilizing agent for the nanoparticles formed in an aqueous medium [18]. The research indicated that when using the ratio of 1 mol of gold salt to 2 mol of sodium citrate, gold nanoparticles with a diameter of around 20 nm and a spherical shape are obtained [36]. This is similar to the diameter of the AuNPs (approximately 20 nm) produced using 2% acetic acid at 60 °C

**Figure 3:**
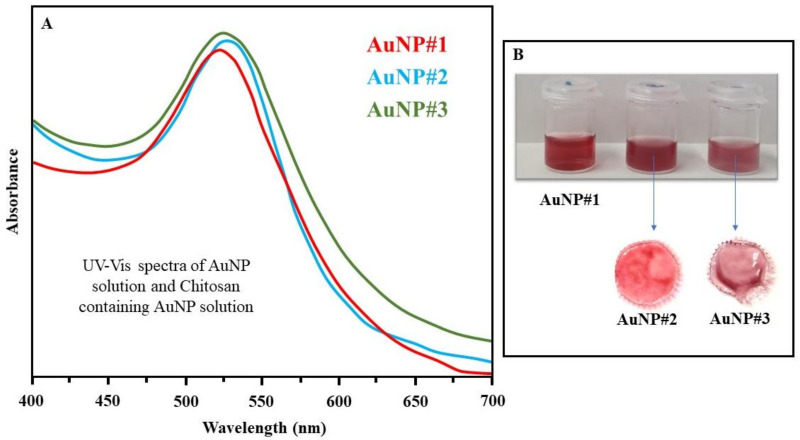
(**A**) UV-vis spectra of solutions AuNP#9, AuNP#10 and AuNP#11. (**B**) Image of solutions AuNP#9, AuNP#10, and AuNP#11 and drop-cast films formed from AuNP#9 and AuNP#11 mixed with tea tree oil.

The solutions of AuNP#9, AuNP#10, and AuNP#11 were analyzed by UV-Vis, and the spectra can be seen in Figure 3A. The red curve represents the absorption spectrum of the gold nanoparticles in the absence of chitosan. It is possible to observe that the maximum absorption is around 520 nm, suggesting spherical gold nanoparticles with a diameter of around 20 nm, as reported in the literature [36,37]. The blue curve refers to solution AuNP#9, while the green one corresponds to solution AuNP#11, with maximum absorptions around 530 and 525 nm, respectively. The shift to higher wavelength values in comparison to solution AuNP#10 (red curve) indicates that the nanoparticles formed may have larger diameters. This shift is expected because when chitosan is added to the solution, the polymer molecules tend to contribute to the stabilization of the formed nanoparticles, causing them to move to locations where there are only citrate molecules. Molecules containing functional groups, such as chitosan, can also act as stabilizing agents for gold nanoparticles. It is believed that the stabilizing effect of chitosan molecules is due to the presence of OH groups, which interact with gold atoms [43]. The color of the solutions is shown in Figure 3B. It is possible to observe that there is a slight difference in color among the solutions, also indicating changes in the size of the gold nanoparticles formed.

### 3.2. Formation of Films and Controlled Release Experiment

The films based on chitosan and gold nanoparticles containing tea tree oil were obtained using the drop-casting technique. Figure 3B shows the films and the corresponding solutions. It is seen that the presence of tea tree oil did not change the system color. Even though both had a similar property, i.e., they did not show solubility in water during the controlled release experiments, their behavior was different. The film obtained from solution AuNP#11 was completely removed after the end of the experiment, suffering only from swelling, while that obtained from the solution AuNP#9 broke into very small pieces.

**Figure 4:**
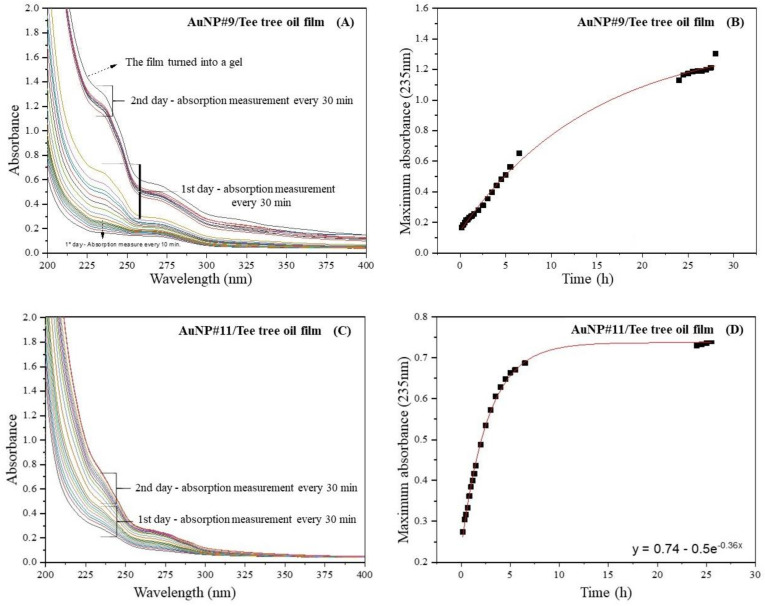
(**A**) UV-vis spectra of the release process of tea tree oil from the AuNP#2- film; (**B**) absorbance maxima at 235 nm of the release process from the AuNP#9-film; (**C**) UV-vis spectra of the release process of tea tree oil from the AuNP#11-film; and (**D**) absorbance maxima at 235 nm of the release process from the AuNP#11-film.

A controlled release process of tea tree oil from the AuNP#9- and AuNP#11-films can be observed in the spectra of Figure 4. It is seen that the maximum of the absorbance band at 235 nm increased with each measurement until the film started to turn into a gel (Figure 4A); the absorption band around 235 nm is related to oil release, as reported by Jiang et al. [44]. The pieces of the film were removed from the water and dried in an oven at 40 °C overnight to compare the mass before and after the release process. The initial mass of the film was (0.11 ± 0.05) g, and it then decreased to (0.05 ± 0.01) g at the end of the experiment, corresponding to a mass loss of (50 ± 5)%. The initial thickness of the AuNP#9 film was (0.055 ± 0.003) mm.

Figure 4B displays the absorbance maxima of the UV-vis spectra (Figure 4A) at a wavelength of 230 nm plotted against the time. As can be seen, the variation of the maximum absorbance with time was more pronounced on the second day. This happened because the film turned into a gel during the release process, which resulted in a massive increase in oil concentration in the solution. Due to the loss of film integrity, the proposed mathematical model did not correctly describe the release behavior, as we can see in the graph in Figure 4B, and, because of the destruction of the film, it was not possible to calculate the diffusion coefficient for this system. As reported by Fu et al. [5], polysaccharides such as chitosan can undergo dissolution in an aqueous medium thanks to the processes of solvent penetration, swelling and untangling, and the relaxation of the polymer chain. When this occurs, the transport of smaller molecules will be conducted by diffusion and/or dissolution.

Through the calibration curve that relates the oil concentration to the maximum absorption at a wavelength of 235 nm, it was possible to obtain Equation (2):

(2) y=0.052+8.21x.

where *x* is the concentration (g/L) and y is the absorbance value. For the film obtained from the AuNP#9 solution, the oil concentration at the end of the release was approximately 0.15 g/L.

Different from what was observed for the film obtained from AuNP#9, the films obtained from AuNP#11 did not transform into a gel, maintaining the integrity of the polymer matrix. A similar experiment was carried out with the AuNP#11-film, and the results are displayed in Figure 4C,D. The maximum of the absorbance band at 235 nm increased with each measurement until an equilibrium was reached after 24 h, and the concentration of the oil released was about 0.8 g/L. Then, four additional spectra were measured every 30 min to confirm that there was no further increase in absorbance. A controlled release over 24 h was observed using the film created with the solution of AuNP#11 as a carrier. Following the measurement of the UV-vis spectra, the film was removed from the water and dried in an oven at 40 °C overnight to compare the mass before and after the release process. The initial mass of this film was (0.15 ± 0.05) g, and then it decreased to (0.05 ± 0.03) g at the end of the experiment, corresponding to a mass loss of about (54 ± 3)%.

Since the release process of tea tree oil from the chitosan matrix is diffusion-controlled, it was described by Fick’s Law, according to Equation (1) [5]. Thus, an exponential function was used to mathematically describe the controlled release. The data are fit best by the exponential function given in Figure 4D. The diffusion coefficient *D* is calculated according to Equation (3)

(3) $$\frac{M_{t}}{M_{0}}=4{\left(\frac{Dt}{\pi h^{2}}\right)}^{2}\;\to \;D=\left(\frac{h^{2}K}{\pi^{2}}\right)$$

with *K* = 0.36 and *h* = 0.054 mm. The resulting diffusion coefficient is 1.1 × 10^−4^ mm^2^/s. 

Initial exploratory studies were carried out in an attempt to obtain chitosan films for the study of the controlled release of tea tree oil. It was observed that in the first 10 min of testing, all of the the oil had already been released, evidencing that chitosan alone does not form a film capable of promoting a controlled oil release system.

### 3.3. Formation of Droplets and Controlled Release Experiment

**Figure 5:**
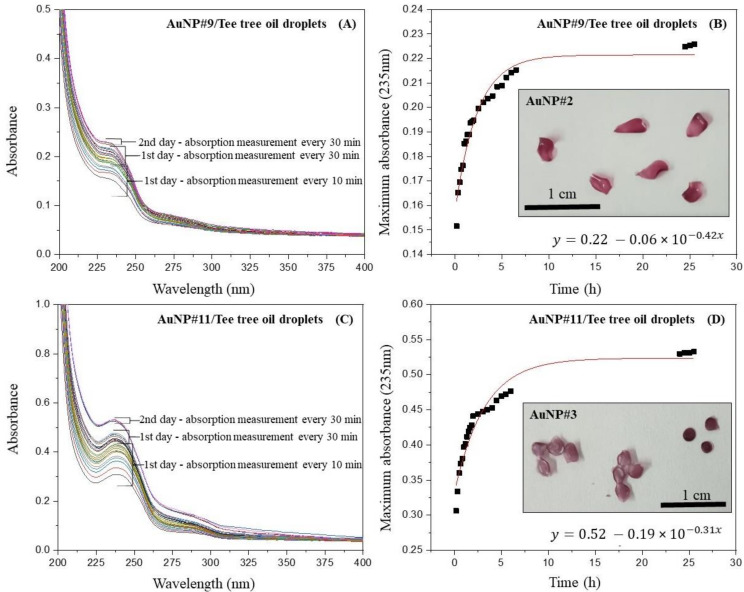
(**A**) UV-vis spectra of the release process of tea tree oil from AuNP#9droplets; (**B**) absorbance maxima at 235 nm of the release process from AuNP#9-droplets; (**C**) UV-vis spectra of the release process of tea tree oil from AuNP#11-droplets; and (**D**) absorbance maxima at 235 nm of the release process from AuNP#11-droplets.

The dry droplets containing chitosan and AuNPs had a diameter of approximately 0.2 cm and a color similar to that found in the films (insets of Figure 5B,D). The droplets obtained from AuNP#9 showed a petal shape, while those obtained from AuNP#11 exhibited a spherical shape. In the insert of Figure 5D, the droplets on the left are partially curved/irregular, resulting in slightly angular folding, which distorts the spherical shape appearance.

The controlled release of tea tree oil present in the droplets was performed in the same way as the release of oil trapped in the films. Figure 5A,C show the UV-vis spectra of the release process of tea tree oil from the droplets, whereas Figure 5B,D show the absorbance maxima of the UV-vis spectra plotted against the time. No release of gold nanoparticles was observed during the process. In the case of droplets obtained from AuNP#9 (Figure 5B), the variation with time seems to be very similar to that shown in Figure 4A,B for the film obtained from AuNP#9, but with a lower limiting value (around 0.53). The initial droplet mass of (0.09 ± 0.01) g decreased to (0.06 ± 0.02) g at the end of the experiment, corresponding to a mass loss of (27 ± 3)%. The thickness of the AuNP#9 droplets was around (0.2 ± 0.05) mm. The diffusion coefficient of tea tree oil in these droplets is *D* = (19 × 10^−4^) mm^2^/s calculated with *K* = 0.42 from the exponential fit of the data. The concentration of oil released was about 0.02 g/L.

In the controlled release process using the AuNP#11-droplets, it was possible to observe that although the film prepared with the same solution started to gel after the first day of the experiment, the droplets remained completely stable and showed no signs of gelling or degradation at all. This can also be seen in the UV-vis spectra since, in contrast to the film, there was no massive increase in absorbance of oil released from droplets after the first day. In addition, the removal of droplets from the water after the experiment was rather simple, while the film was more difficult to remove due to gelling. The gelation process was not observed in the film obtained from the AuNP#9 solution, probably due to the presence of sodium citrate, which promotes a kind of crosslink between the chitosan molecules [45]. Crosslinking brings the polymer molecules closer to each other, increasing their intermolecular interaction and decreasing their affinity for water. The initial mass of the AuNP#11 droplets was (0.10 ± 0.03) g, and then it decreased to (0.08 ± 0.02) g at the end of the experiment, corresponding to a mass loss of (17 ± 4)%. Figure 5D shows the absorbance maxima of the UV-vis spectra plotted against the time. The variation with time seems very similar to that shown in Figure 4B and Figure 5B for the AuNP#11 film and droplets. The thickness of the AuNP#11 droplets was (0.19 ± 0.05) mm, determined using a micrometer gauge. The diffusion coefficient of tea tree oil in these droplets is 12 × 10^−4^ mm^2^/s. The concentration of oil released was about 0.06 g/L.

By comparing the results obtained with AuNP#9- and AuNP#11 droplets, it is possible to conclude that in the data for AuNP#9, a limiting absorbance value of 0.23 is reached after around 5 h. However, the data for AuNP#11 start at an absorbance of more than twice as high and stabilize after around 8 h, when a limiting value of around 0.53 is approximated asymptotically. Table 2 summarizes all the results from the controlled release experiments using films and droplets obtained from both solutions.

The release process begins with the tea tree oil trapped within the polymer matrix. Chitosan also has hydrophilic and hydrophobic regions in its chains, so it will present intermolecular interactions with TTO molecules. As it is initially trapped, to be released, the oil needs to move from the inside through the polymer carrier to the interface between the carrier and the release medium, which is water. As soon as it reaches the solid/liquid interface, the final step of diffusion is to move the oil out of the polymer carrier and into the water. Although this process might seem simple, it is affected by numerous complex factors, such as the structural characteristics of the chitosan matrix and the physico-chemical properties of the solute and the release environment. Fu et al. [3] describe the transport mechanism involving the release of components through polymeric matrices. Among the different systems described, they showed that when there is no destruction of the polymer during the release process, the substance is eliminated through a diffusion process, in which it is possible to obtain the diffusion coefficient of the system through derivations of the second law of Fick diffusion. In the case of matrices obtained from chitosan, as the material does not degrade during the release process, the conditions used for the preparation of the films and droplets were safe to preserve the integrity of the polymer and its components, the main driving forces for oil release may be associated with solute diffusion and the swelling of the polymer matrix [3]. In the case of droplets, due to the treatment in TPP solution, which enhanced crosslinking between the chitosan polymeric chains, the swelling is not very accentuated, causing the main driving force of the release to be the diffusion of the solute itself.

## 4. Conclusions

The incorporation of AuNPs into a chitosan matrix to create drop-cast films and droplets was possible through two different approaches: the formation of nanoparticles inside a chitosan solution in the absence of citrate and the combination of a chitosan solution with an AuNP solution (with sodium citrate) created separately. The experiments showed that the citrate influenced the film stability in water since the film without this agent turned into a gel. The droplets, on the other hand, remained intact during the controlled release experiments, regardless of the presence of citrate during the formation of gold nanoparticles. The release time of the melaleuca oil was around 25 h for the films and for the droplets, which possibly indicates that in addition to chitosan, AuNPs interact with tea tree oil within the polymer matrix, delaying the release process. Studies involving the synthesis of gold nanoparticles in the presence of chitosan revealed that both the concentration of acetic acid in the solution and the reaction temperature of the formation process influenced the size and shape of AuNPs. Indeed, a higher temperature (60 °C) led to spherical-like particles, and lower temperatures favored the formation of hexagonal and trigonal particles. 

## Figures and Tables

**Table 1:** Preparation of AuNPs in chitosan using different parameters.

**Sample**	**#1**	**#2**	**#3**	**#4**	**#5**	**#6**	**#7**	**#8**	**#9**
**Acetic acid concentration**	0.5%	1%	2%	0.5%	1%	2%	0.5%	1%	2%
**Temperature**	RT	RT	RT	40 °C	40 °C	40 °C	60 °C	60 °C	60 °C

**Table 2:** Summary of the controlled release results of AuNP#11- and AuNP#9-films and droplets.

**Sample**	**Initial Mass [g]**	**Thickness [mm]**	**Δm (%)**	$\mathrm{Diffusion}\;\mathrm{Coefficient}\;\left[\frac{mm^{2}}{s}\right]$	Oil Released Concentratio [g/L]
**Film**					
AuNP#9	0.11 ± 0.05	0.055 ± 0.003	50 ± 5	________	0.15
AuNP#11	0.15 ± 0.05	0.054 ± 0.002	54 ± 3	1.1 × 10^−4^	0.08
**Droplets**					
AuNP#9	0.09 ± 0.01	0.20 ±0.05	27 ± 3	1.23 × 10^−3^	0.02
AuNP#11	0.10 ± 0.03	0.19 ± 0.05	17 ± 4	19 × 10^−4^	0.06

## Data Availability

The data presented in this study are available on request from the corresponding author.
